# Constancy of the anterior lateral malleolar artery anastomosis in retrograde lateral supramalleolar flaps: Anatomical basis and clinical relevance

**DOI:** 10.1016/j.jpra.2026.01.040

**Published:** 2026-02-03

**Authors:** Thach Ngoc Nguyen, Thi Cao

**Affiliations:** a Hospital for Traumatology and Orthopaedics, Ho Chi Minh City, 929 Tran Hung Dao Street, Ward Cho Quan, Ho Chi Minh City, Vietnam; b Department of Orthopedics and Rehabilitation, School of Medicine, University of Medicine and Pharmacy at Ho Chi Minh City, 217 Hong Bang Street, Ward Cho Lon, Ho Chi Minh City, Vietnam

**Keywords:** Anterior lateral malleolar artery, Foot reconstruction, Soft tissue defect, Retrograde lateral supramalleolar flap, Vascular anatomy

## Abstract

**Background:**

The lateral supramalleolar (LSM) flap is traditionally based on peroneal perforators, but its retrograde vascularization via the anterior lateral malleolar artery (ALMA) remains insufficiently defined. Clarifying this pathway may expand reconstructive options for dorsal forefoot defects.

**Methods:**

Anatomical dissections were performed on 31 fresh above-knee amputated limbs, focusing on the retrograde anastomosis between the ALMA and the descending branch of the peroneal artery. Distances, diameters, and perfusion were documented by direct measurements and contrast radiography. Surface landmarks were established: landmark 1 (ALMA origin at the anterior tibial artery–malleolar line intersection) and landmark 2 (fourth metatarsal axis–malleolar line intersection). Clinical validation was conducted in 38 patients with dorsal forefoot defects reconstructed using retrograde LSM flaps.

**Results:**

Anastomoses between the ALMA and peroneal artery were consistently identified. The mean distance from landmark 2 to the anastomosis was 13.1 mm, with a mean arterial diameter of 0.8 mm; the ALMA origin was 12.2 mm from landmark 1 with a diameter of 1.0 mm. Flap length averaged 19.8 cm from the anastomosis. Radiography confirmed perfusion extending to the midline of the leg. Clinically, the mean wound size was 7.9 × 4.8 cm, reconstructed with flaps averaging 9.4 × 5.3 cm and 17.4 cm in length. Complete survival was achieved in 35 of 38 cases (92%); three had partial necrosis managed with grafting.

**Conclusion:**

The retrograde ALMA consistently supports the LSM flap, with reproducible anatomical landmarks and reliable perfusion. Clinical outcomes confirm its value as a practical option for dorsal forefoot reconstruction, combining anatomical predictability with high survival rates.

## Introduction

Reconstructing soft tissue defects in the dorsal forefoot presents significant surgical difficulties, primarily because of the scarcity of adjacent donor tissue and the specific requirement for flaps that are thin, flexible, and capable of maintaining both functional and aesthetic integrity. Among the various reconstructive options, the lateral supramalleolar (LSM) flap.^1^, ^2^, ^3^, ^4^, ^5^, ^6^, ^7^, ^8^, ^9^—originally introduced by Masquelet et al.—has become a well-established method for addressing defects in the distal lower limb and foot.^10^ Masquelet identified the consistent presence of the perforating branch of the peroneal artery supplying the skin territory along the lateral border of the lower leg. Based on this vascular anatomy, he introduced the concept of the lateral supramalleolar flap, which can be harvested in either a mixed or reverse flow pattern depending on the vascular configuration.

Subsequent anatomical studies have broadened the understanding of its vascular basis, with most focusing on the perforating branch of the peroneal artery and the inferolateral collateral artery from the anterior tibial system as key contributors.^11^, ^12^, ^13^, ^14^ Despite these advances, the retrograde vascularization through the anterior lateral malleolar artery (ALMA) remains underexplored, particularly in Asian populations. Understanding the exact location, diameter, and consistency of this anastomosis is essential for safe flap harvest, minimizing dissection time, and reducing the risk of complications. Moreover, there is a lack of clinical studies correlating anatomical findings with surgical outcomes in dorsal forefoot reconstruction. This study aims to provide a comprehensive anatomical analysis of the retrograde LSM flap based on the anterior lateral malleolar artery, including vascular mapping, diameter measurements, and radiographic confirmation of perfusion. Additionally, we present clinical results from 38 cases of dorsal forefoot reconstruction using this flap, highlighting the direct application of anatomical knowledge to improve surgical outcomes.

## Materials and methods

### Study design and duration

This study was conducted between April 2021 and January 2025 and consisted of two distinct components: an anatomical investigation and a clinical application.

The anatomical study was performed between April 2021 and April 2024 on 31 fresh above-knee amputated lower limbs obtained following tumor resections. Inclusion criteria were age ≥15 years, preservation of the middle third of the leg down to the entire foot, absence of previous surgical interventions, and no history of peripheral vascular disease.

The clinical application was conducted between December 2021 and January 2025 and included 38 cases in which retrograde lateral supramalleolar flap was used for dorsal forefoot soft-tissue reconstruction. Inclusion criteria were dorsal forefoot defects with exposed tendon or bone on a clean wound bed. Patients with severe crush injuries involving the distal third of the lateral lower-leg soft tissue were excluded.

## Part I. Anatomical study

### Data collection

The following parameters were recorded:

Demographic data (age, gender) of donors.

Location of the anastomosis between the descending branch of the perforating peroneal artery and the anterior lateral malleolar artery (ALMA).

Diameter of the anastomosis.

Origin and diameter of the ALMA.

Distance from predefined surface landmarks to the vascular anastomosis.

• Total length of the lateral supramalleolar flap

The classical boundaries of the lateral supramalleolar flap were defined as originally described by Masquelet: anteriorly by the tibial crest, posteriorly by the posterior border of the fibula, distally by the emergence point of the perforating branch through the interosseous membrane, and proximally by the level halfway up the lower leg.^10^

### Skin landmarks definition

For surgical planning and anatomical orientation, two reproducible surface landmarks were defined. A line connecting the medial and lateral malleoli was established using a plane oriented at a 45-degree angle to the anterior surface of the ankle, with the ankle positioned in neutral alignment.

**Figure 1:**
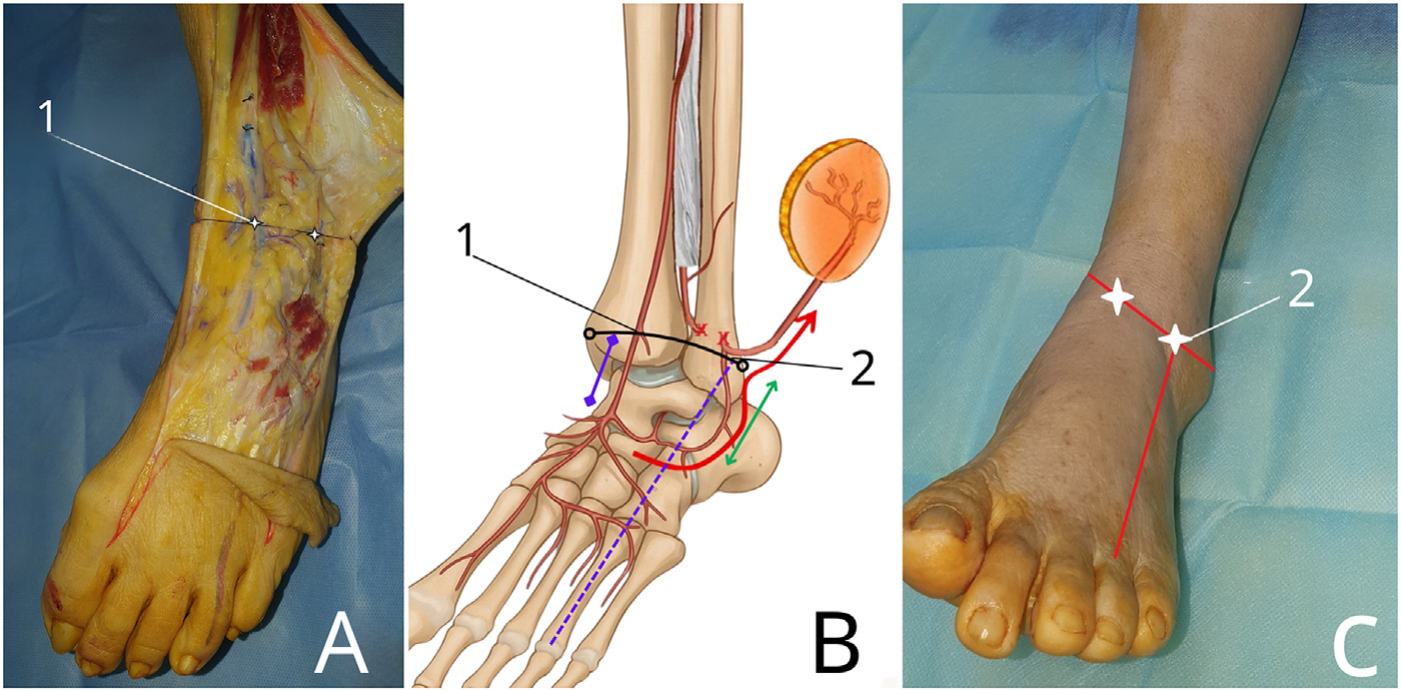
Definition of surface landmarks for retrograde lateral supramalleolar flap planning. (A) Anatomical dissection of the distal lateral leg demonstrating the descending branch of the perforating peroneal artery and its anastomosis with the anterior lateral malleolar artery (ALMA). (B) Schematic illustration showing the vascular anatomy of the retrograde lateral supramalleolar flap, highlighting the retrograde flow through the ALMA after ligation of the perforating peroneal artery. (C) Clinical photograph illustrating the corresponding surface landmarks on the skin. Two reproducible landmarks are defined along a malleolar reference line positioned at a 45-degree angle to the anterior ankle surface: **Landmark 1**, the intersection with the anterior tibial artery; and **Landmark 2**, the intersection with the axis of the fourth metatarsal. These landmarks serve as practical guides for locating the vascular anastomosis and planning safe flap harvest.

• Landmark 1: intersection of this line with the anterior tibial artery.
• Landmark 2: intersection of this line with the axis of the fourth metatarsal bone (Figure 1).

### Flap length measurement

**Figure 2:**
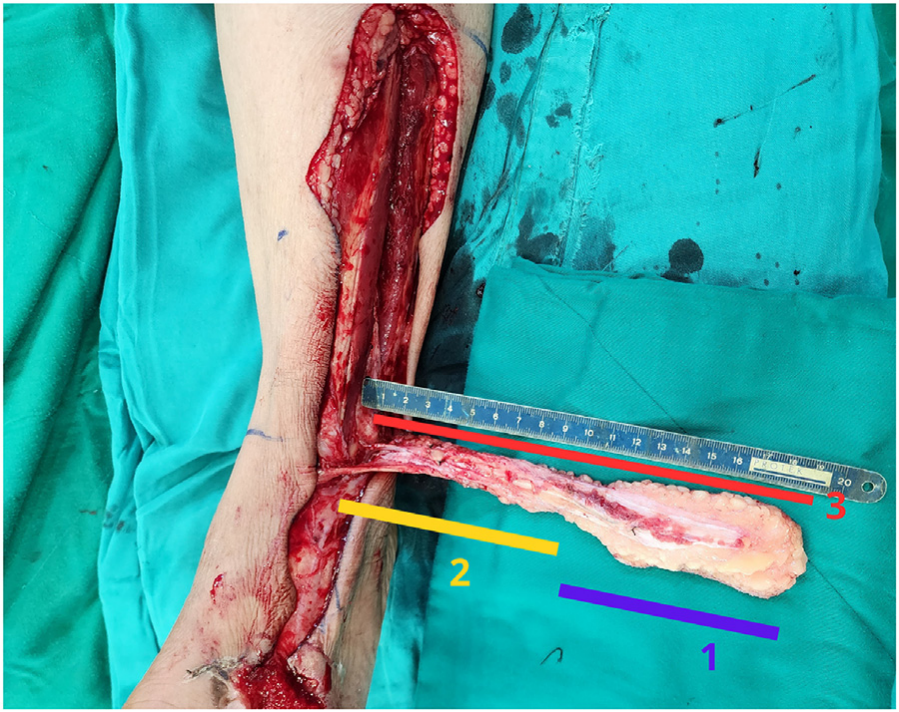
Measurement of total flap length and definition of the pivot point. Illustration demonstrating measurement of the retrograde lateral supramalleolar flap length. Label 1 indicates the length of the skin island, Label 2 represents the adipofascial pedicle length, and Label 3 indicates the total flap length measured from the pivot point to the most distal margin of the skin island along the longitudinal axis of the flap. This measurement defines the maximal arc of rotation of the retrograde flap and provides a practical anatomical basis for pivot point selection and flap length planning.

The total length of the lateral supramalleolar flap was measured from the anastomotic point to the most distal margin of the skin island along the flap axis. This measurement was used to determine the maximal extension potential of the flap (Figure 2).

### Anatomical dissection technique

The lower limb was positioned supine with the ankle maintained at a right angle. Skin landmarks corresponding to landmarks 1 and 2 were marked. A longitudinal incision was made along the tibial crest to expose and transect the anterior tibial artery. Ten milliliters of a 1:10,000 diluted methylene blue solution were injected to visualize arterial pathways. The perforating branch of the peroneal artery was identified at its emergence through the interosseous membrane and ligated at that point.

**Figure 3:**
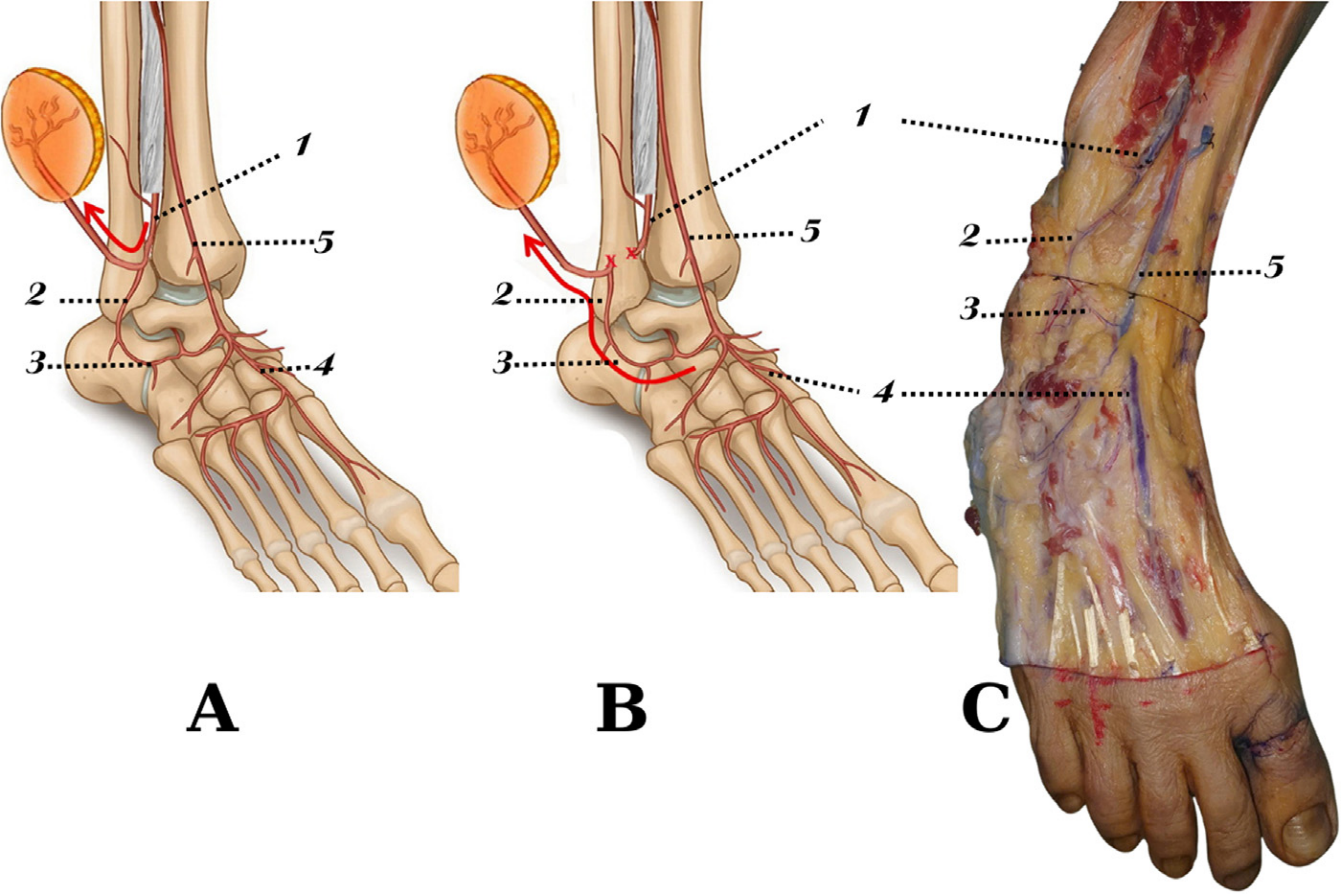
Vascular basis of the retrograde lateral supramalleolar flap. (A) Schematic illustration of the classical mixed-flow pattern of the lateral supramalleolar flap based on the perforating branch of the peroneal artery. (B) Schematic illustration of the retrograde-flow configuration following ligation of the perforating branch, illustrating retrograde perfusion through the anterior lateral malleolar artery. (C) Anatomical dissection illustrating a representative anastomotic connection between the descending branch of the perforating peroneal artery and the anterior lateral malleolar artery. This figure illustrates the anatomical rationale underlying the retrograde vascular supply of the lateral supramalleolar flap. 1: Peroneal artery (interosseous membrane incised for exposure); 2: Descending branch; 3: Anterior lateral malleolar artery; 4: Dorsalis pedis artery; 5: Anterior tibial artery.

The perforating deep fascial branch supplying the skin flap was then dissected and traced distally as the descending branch. The anastomosis between the descending branch and the ALMA was identified, and arterial diameters were measured. The location of the anastomosis was documented in relation to the predefined surface landmarks (Figure 3). The total flap length was recorded.

### Radiographic assessment

Following anatomical dissection, 50 mL of contrast agent was injected at the same arterial site used for methylene blue injection. The midline of the lower leg was marked on the skin to assess the extent of vascular perfusion. The skin and subcutaneous tissue over the lateral distal leg were elevated, and radiographic imaging was performed to visualize the vascular network and distal perfusion territory of the flap.

## Part II. Clinical application

### Clinical data collection

The following clinical parameters were recorded:

Patient demographic data (age, gender)

Wound size

Harvested flap size

Total flap length

Flap survival rate

### Surgical technique

Patients were placed supine with a bolster under the ipsilateral hip to internally rotate the leg. The wound was re-debrided before flap elevation. The flap axis was drawn along the lateral distal leg between the anterior tibial crest and the posterior fibular border. The pivot point was identified preoperatively, and an additional 2 cm was added to the arc of rotation to avoid acute angulation. The flap was designed approximately 20% larger than the defect to compensate for soft-tissue contraction.

Flap elevation was initiated distally in a subfascial plane using a modified adipofascial pedicle technique. The perforating branch of the peroneal artery and its descending branch were identified, and retrograde perfusion through the anastomosis with the anterior lateral malleolar artery was confirmed. The anterior perforating branch of the peroneal artery was ligated to establish a retrograde-flow flap.

Depending on local conditions, the pedicle was transferred through a wide subcutaneous tunnel or positioned beneath a released skin bridge to minimize compression. The flap was rotated to the recipient site and inset without tension. Donor sites were closed primarily when feasible or covered with split-thickness skin grafts.

## Evaluation criteria

### Anatomical study

#### Primary outcomes included

Location and diameter of the anastomosis between the descending branch and the ALMA.

Origin and diameter of the ALMA.

Total flap length measured from both the anastomotic point and the artery origin.

Radiographic assessment of flap perfusion relative to the midline of the lower leg.

#### Clinical study

The primary outcome was flap survival (complete survival or partial necrosis). Secondary outcomes included wound size, flap size, total harvested flap length, and postoperative complications such as infection, wound dehiscence, or need for secondary intervention.

#### Statistical analysis

All data were recorded and analyzed using Stata version 16.0. Descriptive statistics were used to summarize the data. Continuous variables, including distances, diameters, and flap lengths, were reported as means ± standard deviations (SD) and ranges. Categorical variables, such as flap survival rates and complication rates, were presented as frequencies and percentages.

Comparisons between anatomical measurements and clinical flap dimensions were described to validate the anatomical findings. As this was a descriptive and observational study, no inferential statistical tests were performed. A *p*-value of < 0.05 was considered statistically significant where applicable.

## Results

### Anatomical study

A total of 31 lower limbs were dissected, including 17 male and 14 female specimens. The age of the donors ranged from 15 to 78 years, with a mean age of 38.1 ± 21.1 years.

Retrograde vascular supply to the lateral supramalleolar flap via an anastomosis between the anterior lateral malleolar artery and the descending branch of the peroneal artery was consistently identified in all specimens (Table 1). The distance from the anastomosis site to the anterior lateral malleolar artery averaged 8.5 ± 5.6 mm (range: 1.7–19.4 mm).

**Table 1:** Data on flap length and vascular diameter (*n* = 31).

Order number	Total flap length (cm)		Arterial diameter (mm)	
	The farthest point along the axis of the flap to		Measured at the junction of the anterior lateral malleolar artery	
	The junction of the anterior lateral malleolar artery and the descending branch	The origin of the anterior lateral malleolar artery	Anastomosing with the descending branch	The origin of the anterior lateral malleolar artery
1	27.1	29.1	0.7	1
2	16.8	19.9	0.9	0.9
3	17.4	19.4	0.9	0.9
4	18.5	21.4	1	1
5	20.8	21.8	0.7	1
6	21.7	22.9	0.7	1.2
7	21.1	23	1	0.9
8	20.1	21.8	1	0.9
9	20.3	19.6	0.7	1
10	20.6	21.7	0.7	1.1
11	20.8	22.2	1	1
12	19.2	21.4	1	1.5
13	21.2	22.5	0.8	1.3
14	16.7	19.4	1	1
15	21.4	24.1	0.6	0.7
16	21.2	22.6	0.6	1
17	17.2	21.1	0.5	0.6
18	21.8	23.3	1	0.9
19	17.1	20.1	1	0.9
20	19.9	23.4	0.8	0.9
21	16.8	18.6	0.7	0.9
22	20.3	23.9	0.6	0.8
23	17	19.9	0.8	1
24	22.1	25	0.7	0.8
25	17.1	18.7	0.8	0.9
26	17	19.6	0.7	0.9
27	27.4	30.3	0.7	0.7
28	16.1	19.4	0.6	0.8
29	19	20.3	1	0.9
30	21.1	24	0.6	0.6
31	18.2	19.6	0.6	0.8

The vascular diameters were measured as follows: the anastomosis between the descending branch and the anterior lateral malleolar artery had an average diameter of 0.8 ± 0.2 mm (range: 0.5–1.0 mm), while the origin of the anterior lateral malleolar artery had an average diameter of 1.0 ± 0.2 mm (range: 0.6–1.5 mm) (Table 1).

The average distance from landmark 1 (the intersection with the anterior tibial artery) to the artery origin was 12.2 ± 4.9 mm (range: 3.7–23.1 mm). The distance from landmark 2 (the intersection with the fourth metatarsal axis) to the anastomosis site was 13.1 ± 7.3 mm (range: 1.2–27.7 mm) (Table 2).

**Table 2:** Technical data in the study.

	Anastomosis location	
Variable	descending branch with the anterior lateral malleolar artery	the origin of anterior lateral malleolar artery
Landmark on the skin (*n* = 31) Landmark 1	Average distance (mm)	Average distance (mm)
Landmark 2	13.1 ± 7.3 (from 1.2 to 27.7)	12.2 ± 4.9 (from 3.7 to 23.1)
Average arterial diameter (mm)	0.8 ± 0.2 (from 0.5 to 1)	1 ± 0.2 (from 0.6 to 1.5)

The total length of the lateral supramalleolar flap, measured from the anastomosis point, was 19.8 ± 2.8 cm (range: 16.1–27.4 cm). When measured from the origin of the anterior lateral malleolar artery, the flap length was 21.9 ± 2.7 cm (range: 18.6–30.1 cm).

**Figure 4:**
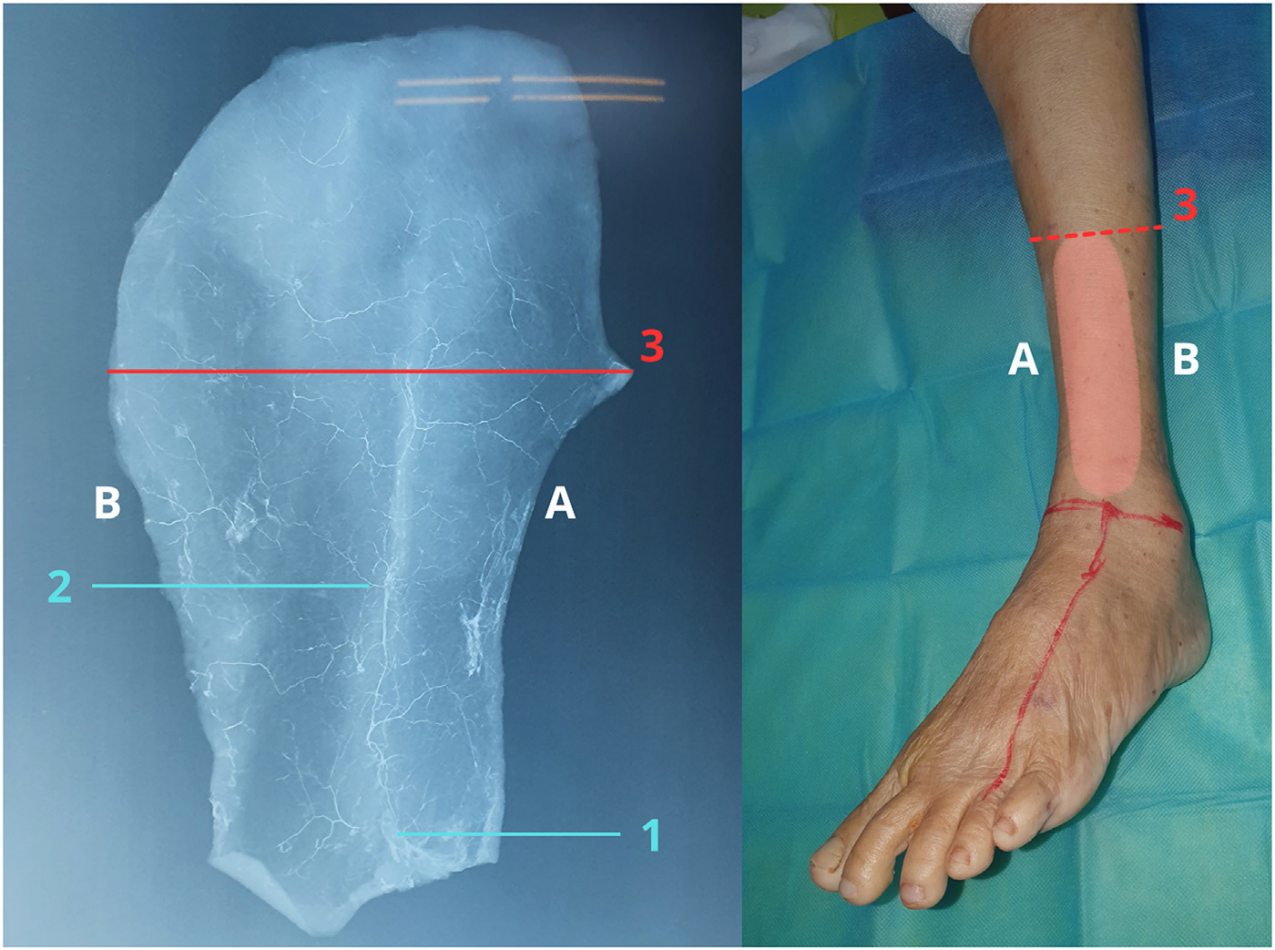
Radiographic validation of flap perfusion territory. Radiographic imaging following contrast injection demonstrates a continuous vascular network supplying the retrograde lateral supramalleolar flap. Perfusion is observed extending toward the midline of the lower leg, illustrating the distribution of retrograde blood flow through the anterior lateral malleolar artery. (A) Tibial crest side. (B) Posterior aspect of the fibular border. The red reference line indicates the midline of the lower leg. Label 1 marks the point where a deep fascial perforator enters and contributes to the vascular network within the skin flap, Label 2 indicates the vascular axis of the skin flap, and Label 3 denotes the position of the midline of the lower leg. These radiographic findings provide imaging-based support for the anatomical feasibility of harvesting retrograde lateral supramalleolar flaps.

Radiographic imaging confirmed that all flaps demonstrated a vascular network reaching the midline of the lower leg (Figure 4).

### Clinical application

**Figure 5:**
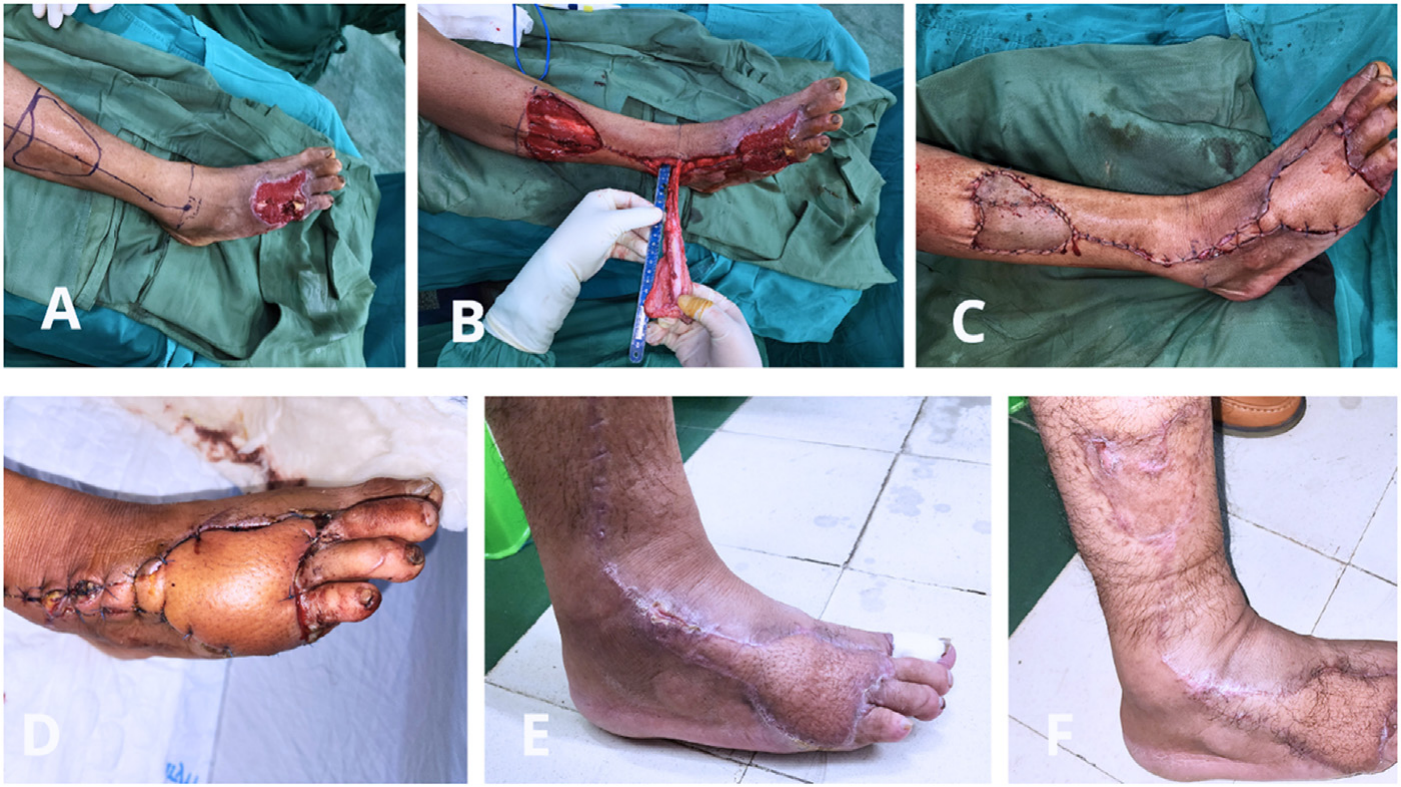
Clinical application of the retrograde lateral supramalleolar flap. (A) Preoperative appearance of a dorsal forefoot soft-tissue defect with exposed bone. (B) Intraoperative elevation of the retrograde lateral supramalleolar flap with an adipofascial pedicle based on the anterior lateral malleolar artery, demonstrating measurement of the total flap length obtained to ensure adequate reach for complete coverage of the defect. C) Immediate postoperative appearance after flap inset and contour adaptation to the recipient site. (D) Early postoperative view demonstrating flap viability. (E, F) Long-term follow-up showing stable coverage, acceptable contour, and satisfactory aesthetic outcome. This figure demonstrates the stepwise surgical application of the retrograde lateral supramalleolar flap and highlights its effectiveness in dorsal forefoot reconstruction.

In the clinical series, 38 retrograde lateral supramalleolar flaps were performed to reconstruct dorsal forefoot soft tissue defects (Figure 5). The mean wound size was 7.9 ± 3.0 cm × 4.8 ± 1.5 cm. The mean flap size harvested was 9.4 ± 3.0 cm × 5.3 ± 1.4 cm. The average total flap length obtained was 17.4 ± 3.0 cm (range: 10–24 cm).

Of the 38 flaps, 35 achieved complete survival. Three flaps developed partial necrosis involving less than 50% of the flap area, which required secondary debridement and skin grafting. All these cases subsequently healed without further complications. The overall flap success rate was 92%.

## Discussion

The present study provides new anatomical and clinical insights into the retrograde lateral supramalleolar (LSM) flap, particularly its vascular basis via the anterior lateral malleolar artery (ALMA). Three major findings emerged: (1) a consistent anastomosis between the ALMA and the descending branch of the peroneal artery was observed in all specimens; (2) radiographic perfusion studies demonstrated reliable vascular extension to the midline of the leg, supporting safe flap elevation even in long designs; and (3) clinical validation in 38 reconstructions showed a 92% overall survival rate, confirming the practical reliability of the flap for dorsal forefoot defects.

Previous studies by Masquelet et al. mainly focused on the perforating branch of the peroneal artery as the primary blood supply.^11^ Le Nen et al. and Kai Rong et al. expanded this understanding by highlighting the role of the inferolateral collateral artery in providing an alternative vascular pathway.^12^^,^^14^ However, few studies have specifically focused on the anterior lateral malleolar artery and its retrograde contribution to the LSM flap. Our findings address this gap. In addition to confirming the consistency of the vascular anatomy, this study provides precise data regarding the location and size of the vascular anastomoses. The average distance from landmark 2—the intersection point of the fourth metatarsal axis with the malleolar line—to the anastomosis site was 13.1 ± 7.3 mm, with an arterial diameter at the anastomosis of 0.8 ± 0.2 mm. Moreover, the origin of the anterior lateral malleolar artery was located an average of 12.2 ± 4.9 mm from landmark 1, with an arterial diameter of 1 ± 0.2 mm. These measurements are directly applicable in preoperative mapping and surgical planning, providing reliable guidance for flap design and safe dissection.

Radiographic imaging confirmed perfusion extending to the midline, validating that the retrograde ALMA pathway can safely nourish larger skin paddles. This correlates with the clinical observation that harvested flaps up to 24 cm in length survived, parallel to the maximum anatomical length of 27.4 cm. This finding highlights a key benefit of basing the lateral supramalleolar flap on the anterior lateral malleolar artery, as the retrograde ALMA inflow effectively increases the distal reach of the flap while maintaining reliable perfusion. In practical terms, this vascular configuration allows safe coverage of more distal forefoot defects without compromising flap viability. Such correlation between anatomy and clinical outcome has been seldom reported in earlier literature. Goil et al. and Nguyen et al. described the clinical utility of the LSM flap, but without detailed anatomical correlation.^8^^,^^15^^,^^16^ Our study bridges this gap by showing that radiographic confirmation of perfusion directly translates into surgical safety, particularly in extended flap designs.

Clinically, the retrograde LSM flap has proven to be a practical and reliable reconstructive option for dorsal forefoot defects, where local flaps are often insufficient and free flaps are technically demanding. Its reliability is directly supported by the anatomical findings of this study. By utilizing the anterior lateral malleolar artery as the pivot point, we achieved coverage of defects without the need for microvascular anastomosis, reducing operative complexity and hospital stay. The high survival rate in our series, combined with anatomical consistency, demonstrates that the retrograde LSM flap is not only theoretically sound but also clinically effective. In our clinical application, 38 flaps were harvested following the anatomical landmarks identified from dissection, allowing precise planning of flap size and pivot point. Our 92% survival rate highlights the advantage of precise anatomical mapping combined with careful flap harvest. The average flap length harvested (17.4 cm) was consistent with the measured anatomical length (19.8 ± 2.8 cm), confirming the validity of using surface landmarks for surgical design. Partial flap necrosis occurred in three patients (aged 29, 50, and 51 years), with flap lengths ranging from 14 to 19 cm. All cases were preceded by postoperative venous congestion, suggesting compromised venous outflow rather than arterial insufficiency. Two flaps were transferred through a subcutaneous tunnel, raising the possibility of pedicle compression, while one case required skin-bridge release but still developed venous congestion, likely related to limited venous drainage capacity in relatively long flaps. Additional contributing factors may include excessive tension or kinking at the pivot point, tight wound closure, and postoperative edema. Although no overt intraoperative pedicle injury was identified, subtle handling-related trauma cannot be entirely excluded. These findings emphasize the importance of careful pedicle handling, tension-free flap inset, appropriate selection of the pivot point, and close postoperative monitoring to minimize the risk of partial flap necrosis.

## Limitations

The anatomical analysis was performed on a limited number of fresh cadaveric specimens from above-knee amputations, which may not fully represent the general population. Additionally, venous drainage and the role of the superficial peroneal nerve in flap viability were not investigated. These factors may influence long-term outcomes and warrant further study. Future research should include larger sample sizes and consider Doppler ultrasonography or three-dimensional imaging for better preoperative planning. Prospective multicenter clinical trials are also needed to validate these findings and further refine the indications for the retrograde LSM flap in foot reconstruction.

## Conclusion

This study provides comprehensive anatomical evidence supporting the consistent retrograde vascular supply of the lateral supramalleolar flap through the anastomosis between the anterior lateral malleolar artery and the descending branch of the peroneal artery. By precisely mapping the vascular anatomy and correlating it with clinical outcomes, we demonstrated that this flap is a reliable and versatile option for dorsal forefoot reconstruction. The anatomical landmarks proposed in this study enable safe and reproducible flap harvest, while radiographic validation confirms the flap’s distal perfusion capacity. Our clinical results further reinforce the anatomical findings, establishing the retrograde lateral supramalleolar flap as a first-line choice for covering complex soft tissue defects of the dorsal forefoot with minimal donor site morbidity.

## Other disclosure

All authors declare that the manuscript has not been published elsewhere in any language. All authors are responsible for the accuracy of the results and methods. Each author has carefully read and approved the final version of this manuscript.

## Funding

This study was funded entirely by its authors.

## Ethical approval and patient consent statement

This study was reviewed and approved by an independent institutional ethics committee, in full accordance with the ethical standards of the Declaration of Helsinki. All participants signed consent.

## Declaration of competing interest

All authors have no conflict of interest and any authorship issue had been solved before the manuscript was submitted.

## Appendix Supplementary materials

Image, video 1 dummy alt text

Image, video 2 dummy alt text
